# Margay (*Leopardus wiedii*) in the southernmost Atlantic Forest: Density and activity patterns under different levels of anthropogenic disturbance

**DOI:** 10.1371/journal.pone.0232013

**Published:** 2020-05-06

**Authors:** Paula E. Horn, Maria J. R. Pereira, Tatiane C. Trigo, Eduardo Eizirik, Flávia P. Tirelli

**Affiliations:** 1 Departamento de Zoologia, Programa de Pós-Graduação em Biologia Animal, Instituto de Biociências, Universidade Federal do Rio Grande do Sul, Porto Alegre, Rio Grande do Sul, Brazil; 2 Centre for Environmental and Marine Studies, Universidade de Aveiro, Aveiro, Portugal; 3 Departamento de Biodiversidade, Setor de Mastozoologia, Museu de Ciências Naturais, Secretaria de Meio Ambiente e Infraestrutura, Porto Alegre, Rio Grande do Sul, Brazil; 4 Instituto Pró-Carnívoros, Atibaia, São Paulo, Brazil; 5 Pontifícia Universidade Católica do Rio Grande do Sul (PUCRS), Escola de Ciências da Saúde e da Vida, Porto Alegre, Rio Grande do Sul, Brazil; University of Sydney, AUSTRALIA

## Abstract

The margay (*Leopardus wiedii*) is a small Neotropical arboreal wild cat. This species is thought to be forest-dependent, although few studies so far have directly evaluated the relationships between spatiotemporal aspects of its ecology and landscape characteristics. The aim of this study was to estimate margay population density and activity patterns in six areas with different habitat types and levels of anthropogenic disturbance in the southernmost Atlantic Forest of Brazil. Our working hypothesis was that density and activity patterns differed between areas in response to differences in forest cover and anthropogenic disturbance. Margay records were obtained using camera trapping, during spring and summer from 2017 to 2019. In all areas, the sampling scheme consisted of 20 un-baited stations, set 1km apart, each containing two paired cameras. We assessed the potential effects of environmental variables, including anthropogenic factors, on margay density, rate of detection and space use by comparing nine spatial capture-recapture (SCR) models. Activity patterns of the margay, its potential prey, and competitors were described and compared using the date and time of the records. We obtained 66 records of margay. Two of the six sampled areas were excluded from subsequent analyses due to the small number of records. The density estimated by the top-ranked model varied from 9.6±6.4 individuals/100km^2^ in the area with the highest human disturbance to 37.4±15.1 individuals/100km^2^ in a less disturbed area. Margay densities responded positively to vegetation cover, supporting the hypothesis of forest dependence by the species. Both the margay and their potential prey (small rodents and marsupials) were found to be mostly nocturnal. Margay activity also overlapped with that of the ocelot, *Leopardus pardalis*, and with mammals associated with human presence (wild boar, cattle, domestic dogs and cats). This is the first multi-area study on patterns of density and activity of the margay in the Brazilian Atlantic Forest. We concluded that the margay is mostly nocturnal, and while its densities are positively influenced by forest cover and negatively influenced by human disturbance, the activity pattern of the species does not seem to change across landscapes with distinct levels of human modification. Margay populations seem to be able to persist under moderate levels of habitat modification, highlighting the importance of preserving even small native forest remnants in the highly fragmented Atlantic Forest.

## Introduction

The establishment of appropriate conservation strategies depends on reliable population density information, among other information on species’ ecology [1]. Population size estimates for different areas or time points allows the detection of small and declining populations, geographic range reduction and fragmentation, and vulnerability to human disturbance [2]. These topics are some of the criteria used by IUCN to evaluate if a taxon is under threat [2]. Thus it is crucial to develop studies evaluating population trends [3], viability [4], and status [5]. Additionally, conservation planning should also take into account behavioral aspects of the species in focus. Knowledge on the activity patterns, for example, is relevant to detect i) mechanisms of intra-guild niche segregation which allow species coexistence [6–9], ii) predator-prey interactions [10], iii) thermal preferences and responses to seasonal variation [11], and iv) relationships between the activity pattern and selected environmental variables (*e*.*g*. lunar or artificial lights [12–14] and anthropogenic disturbances [15–17]).

Small and medium-sized South American cats are amongst the least studied felids worldwide [18]. The margay (*Leopardus wiedii*), a small solitary species, is one of the least studied Neotropical felids, although its distribution ranges from northern Mexico to Uruguay and northern Argentina [19–21]. Margay is categorized as “Near Threatened” and declining globally [22]. Margays are possibly the most arboreal of all felids, and thus seem to be strongly dependent on forested habitats [23,24]. Indeed, margays have morphological adaptations that make them excellent climbers, such as long tails that they use for balance and ankles that rotate up to 180° [21,25]. This particular ecological trait suggests that margays maybe more threatened by deforestation than less arboreal species. However, the species’ ecology is poorly known, even in the Atlantic Forest biodiversity hotspot [26], which seems to be one of the areas of highest habitat suitability for the species [24].

Currently, the Atlantic Forest is restricted to small fragments in a matrix of human-dominated landscapes, occupying less than 12% of its original area [27]. This situation suggests that species strongly dependent on forests, such as the margay, may be facing regional extinction in the short term. This may be even more problematic in the southernmost range of the biome, which represents the southern limit of the margay’s distribution [22], where it is expected to naturally occur at lower densities [28,29].

However, density estimates for the margay across its distribution are few, particularly when compared to other felids. Studies carried out in forested areas of Mexico, estimated density ranging between 12 and 81 individuals/100km^2^ [30,31]. According the IUCN [9], there are estimates from Brazil ranging from one to five individuals/km^2^ and up to 15 to 25 individuals/ 100 km^2^; however, the specific locations of the original studies are not available [22,32,33]. These studies, however, used traditional capture-recapture models, which fail to assess the spatial structure of the ecological processes [1]. Spatial capture-recapture (SCR) models should be able to overcome this limitation by incorporating spatial information from the individual detections [1,34,35]. In contrast to density estimates, a much greater number of studies has described activity patterns of margay across its range, suggesting that, overall, the species is nocturnal [6,8,17,30,31,36–39], though in some areas it may show a cathemeral pattern [40].

The first cycle of the Brazilian National Action Plan for the Conservation of Small Cats (CENAP/ICMBio) defined seven specific objectives, one of which is to assess how different natural and anthropogenic processes influence the populations of small felids [41]. To address this objective, here we aimed to estimate population density of margays using spatial capture-recapture models, and to describe its activity patterns across a range of areas with different levels of human disturbance in the southernmost limit of the Atlantic Forest. We hypothesize: i) density differs across study areas; ii) activity pattern is mostly crepuscular/nocturnal but may change across areas. We expect margay densities to respond positively to forest cover and negatively to human disturbance and ocelot (*Leopardus pardalis*) presence, as margays tend to respond to the presence of other felids. In fact, Oliveira et al. (2010) reported an ‘ocelot effect’, suggesting that the presence of the ocelot, negatively impacts densities of margay and other small cats [32]. Additionally, we expect *L*. *wiedii* to present more nocturnal activity in areas with higher levels of anthropogenic modification (with more occurrence of domestic and exotic species) [15,42]. Ultimately, we aim to generate baseline information for the definition of management actions towards margay conservation at the southern extreme of its distribution, where it may be particularly sensitive to population fluctuations [28,29] and is categorized as “Vulnerable” [43,44].

## Materials and methods

### Study area

**Fig 1:**
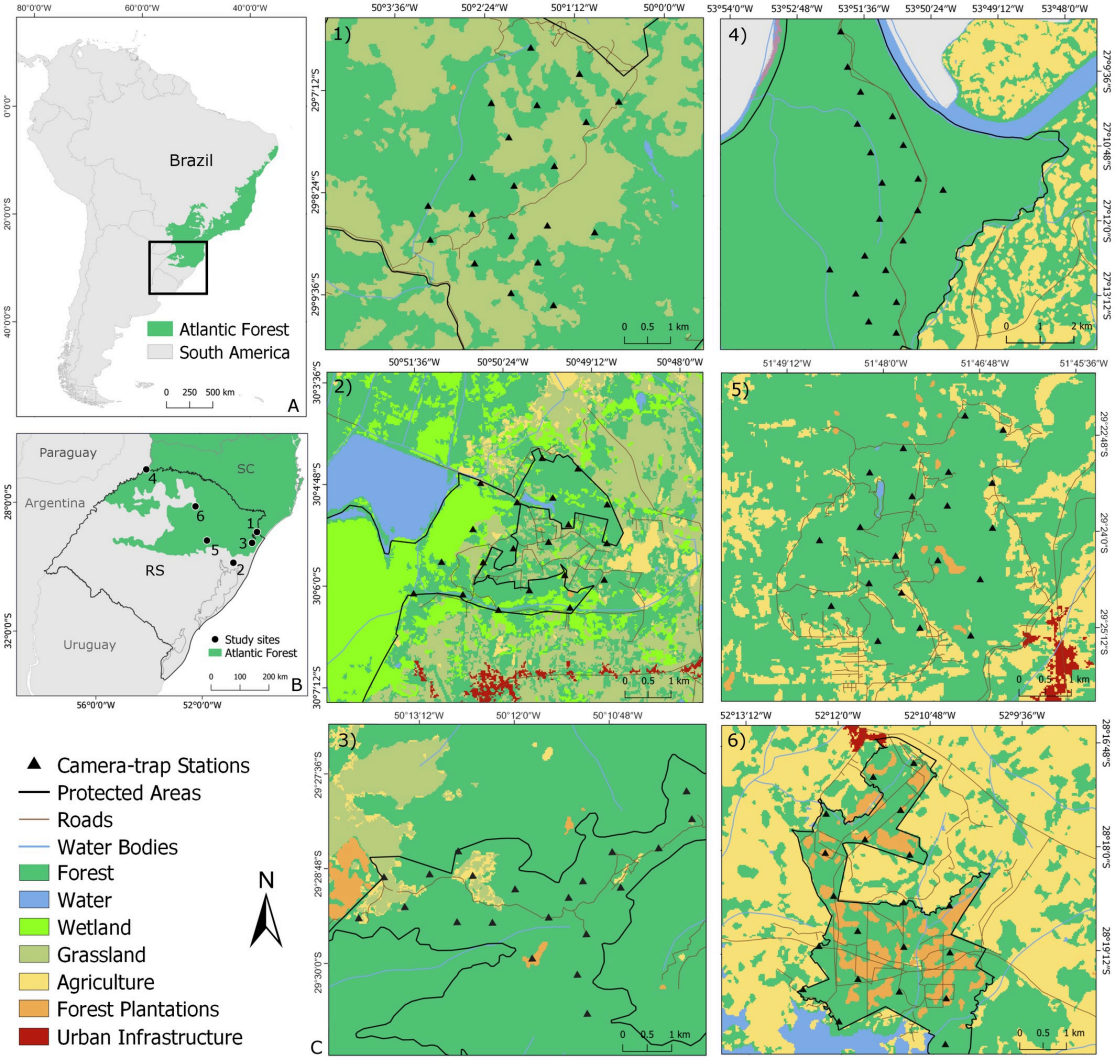
Study area. A) Location of the study area in the southernmost Atlantic Forest in Brazil (in black). B) Location of the six areas in Rio Grande do Sul state (black points) sampled between 2017 and 2019. C) Location of the 20 camera-trap stations in each area (set 1km apart): 1) Serra Geral National Park (SGNP), 2) Banhado dos Pachecos Wildlife Refuge (BPWR), 3) Pró-Mata Center for Research and Conservation of Nature (PROMATA), 4) Turvo State Park (TUSP), 5) Teutônia (TEUT), 6) Passo Fundo National Forest (PFNF).

We sampled six areas in Rio Grande do Sul state, southernmost Brazil (Fig 1). This region comprises the southern portion of the margay’s distribution [22] and the southernmost limit of the Atlantic Forest biome. The Atlantic Forest extends beyond tropical climates, with semi-deciduous and ombrophylus mixed forests (*Araucaria* forests) at higher elevations gradually replacing the dense ombrophylus forests that are typical of the lower altitudes [45–47]. This region also includes the ecotone between the Atlantic Forest and the Brazilian Pampas, which increases the structural and compositional landscape complexity, with changes in climate, elevation, vegetation and, consequently, beta diversity [48–50]. Only 12% of the original area of the Atlantic Forest persists, with the remaining area having been almost completely replaced by croplands and other human-modified landscapes [27]. Indeed, due to habitat loss and fragmentation [22,43] the margay is categorized as “Vulnerable” in Rio Grande do Sul state [43,44].

We collected data from 120 sites in six areas spanning a range of habitat types and human land-use intensity; the six areas were:

1. Serra Geral National Park (SGNP) (29°08’2”S, 49°59’40”W), a federal conservation unit of strict protection, constituted by a mosaic of natural landscapes of different vegetation formations, such as *Araucaria* forests [51]. The park portion sampled in this study was in the process of expropriation, with cattle still roaming in some patches;
2. Banhado dos Pachecos Wildlife Refuge (BPWR) (30°06’22”S, 50°52’11”W), a protected area located close to large urban centers (ca. 28km from the state capital, Porto Alegre); this region is composed of large areas of plains and grasslands [22,52] and forest fragments among roads and residences; two camera-trap stations ended up set outside the protected area and close to human settlements, due the standard distance between the stations in the sampling scheme. This study area is located in the ecotone between the Atlantic Forest and the Pampa biomes [53].
3. Pró-Mata Centre for Research and Conservation of Nature (PROMATA) (29°28’54”S, 50°10’35”W) is a private natural heritage reserve, the largest private protected area in the state [54]; previously occupied by small farms and agropastoral activities, this area has been under natural regeneration for over 20 years [55];
4. Turvo State Park (TUSP) (27°08’44”S, 53°53’10”W), a strict protected area created in 1954, this park is one of the largest conservation units in the state [56] and the most pristine area in this study;
5. A rural area close to the city of Teutônia (TEUT) in the central region of the state (29°26’36”S, 51°47’57”W); this is a non-protected area comprising a matrix of small private properties with different agricultural activities (corn and soy crops) and livestock (chicken, pigs, dairy cattle); in high hills of the region, there are fragments of native and exotic forests that are functionally connected to each other;
6. Passo Fundo National Forest (PFNF), in the northern region of the state (28°18’47”S, 52°10’55”W), is a protected area with sustainable land use, resulting from the restoration of an agricultural area, predominantly planted with native trees [57,58].

These study areas were categorized with respect to different anthropogenic disturbance levels in a parallel study performed simultaneously at the same areas [59]. The authors generated the levels base on different variables (e.g. distance to forest edge, distance to nearest cell phone tower, stable light at night values, predicted abundances of domestic carnivores, etc.). Here we follow the gradient from the most disturbed area to the most preserved one: BPWR, TEUT, PFNF, SGNP, PROMATA, TUSP.

### Field sampling

We collected margay records between 2017 and 2019, during summer and spring, averaging 60-sampling days (56 to 62 days) per area, to assume closed margay populations [60]. In each area we installed 20 camera-trap stations placed ca.1 km apart (Fig 1C). This layout was defined based on the diameter of female home ranges for the species (<1km^2^) estimated in previous studies [22,32]. Each sampling station was composed of two passive infrared digital camera-traps, one at each side of a wildlife trail or road, totaling 40 camera-traps per sampling area. Pairing the cameras allowed recording the two flanks of the detected animals for individual identification through the individual-specific pelage pattern [61]. We set up the unbaited camera traps at 30–40 cm above the ground. We programmed the cameras in video mode (10 s, with a 5 s motion triggered delay), to remain active 24 h per day, recording date and time. We used several camera models and brands: Bushnell Trophy Cam^™^ and Nature View^®^ (Bushnell Outdoor Products, Overland Park, Kansas), Digital Game Camera Moultrie (Moultrie Products, LLC, Birmingham, Alabama), Browning^®^ Trail Cameras (Prometheus Group, LLC, Birmingham, Alabama) and Scout Guard Infrared Digital Scouting Camera (Boly Media Communications Co. Ltd., Shenzhen, China).

### Density and detection covariates

Based on the literature and previous knowledge on margay’s biology, we defined a set of environmental covariates, including anthropogenic-related ones, as predictor variables for the species’ spatial scale, density and rate of detections. We used the individuals’ sex as a covariate to investigate the spatial scale, eight covariates for fitting models for rate of detection, and five covariates for density modeling; we evaluated if the three parameters differed between areas (Table 1). We excluded highly collinear predictors using the variance inflation factor (VIF), excluding variables with VIF > 6 [62](S1 Table).

**Table 1:** Selected covariates and respective predicted effects on spatial-scale, density and rate of detection of the margay cat.

Covariate (units)	Code	Description/ Source	Prediction
***Density***			
**Vegetation cover** (1km^2^)	ndvi	Normalized Difference Vegetation Index Values range from 0 (non-forest) to 1 (dense forest cover) MODIS Product generated by the Land Processes Distributed Active Center (LP-DAAC)[63]	Density will be higher in areas with higher vegetation cover
**Distance to water** (1km^2^)	diswater	Euclidean distance raster created in ArcGis based on shapefile of water bodies of the Regional Executive Organization for Environmental Protection [64] Values range from 0 to 10km	Density will be higher closer to natural water bodies
**Human population density** (1km^2^)	popdens	Estimate of human population density (ranging from 0 to 10000 people per square kilometer) Socioeconomic Data and Applications Center; Gridded Population of the World, Version 4 (GPWv4) for2015 [65]	Density will decrease with increasing human densities
**Distance to roads** (1km^2^)	disroads	Euclidean distance raster created in ArcGis based on shapefile of roads of the Regional Executive Organization for Environmental Protection[64]; missing roads included manually through own observations Values range from 0 to 10km	Density will be higher as one moves further from roads (paved or unpaved roads)
***Rate of Detection***			
**Small mammals** (per hour)	smam	Number of independent detections (>1 h apart) of small mammals (small rodents and marsupials) per site-by-occasion	Rate of detection will increase with the presence of small mammals, potential prey of the species
**Small birds** (per hour)	sbirds	Number of independent detections (>1 h apart) of small birds (Passeriformes) per site-by-occasion	Rate of detection will increase with the presence of small birds, potential prey of the species
**Ocelot** (per hour)	ocelot	Number of independent detections (>1 h apart) of ocelots (*Leopardus pardalis*) per site-by-occasion	Rate of detection will decrease with the presence of ocelot, potential competitor or intraguild predator
**Dogs** (per hour)	dogs	Number of independent detections (>1 h apart) of dogs (*Canis familiaris*) per site-by-occasion	Rate of detection will decrease with the presence of dogs, potential predators of margay
**Cats** (per hour)	cats	Number of independent detections (>1 h apart) of domestic cats (*Felis catus)* per site-by-occasion	Rate of detection will decrease with the presence of cats, potential competitors of margay
**Trigger time** (seconds)	trigger	Trigger speed of each camera-trap brand/model, and time delay necessary for the camera to shoot a picture once an animal has interrupted the infrared beam within the camera’s detection zone (from the manual instructions) ^a^	Minor response time will increase the rate of detection of margay
**PIR detection range** (meters)	pir	Passive Infra-Red (PIR) distance detection range of each camera-trap brand/model (from the manual instructions) ^a^	Higher PIR detection range values, i.e., larger detection zone will increase the rate of detection of margay
***Spatial-scale***			
**Sex**	sex	Sex of the individual (female, male or undetermined)[66]	Spatial use will differ between the sexes
**Sessions**^b^	session	Data from groups which can be sampled in different spatial or temporal independent studies, these data groups are called "sessions"[66]. We used the sampling areas as our sessions	The density will differ between the areas

^a^ we used the setting best camera-trap model to represent the camera-trap station (site).

^b^ we used this covariate for all parameters tested.

### Density modeling

**Fig 2:**
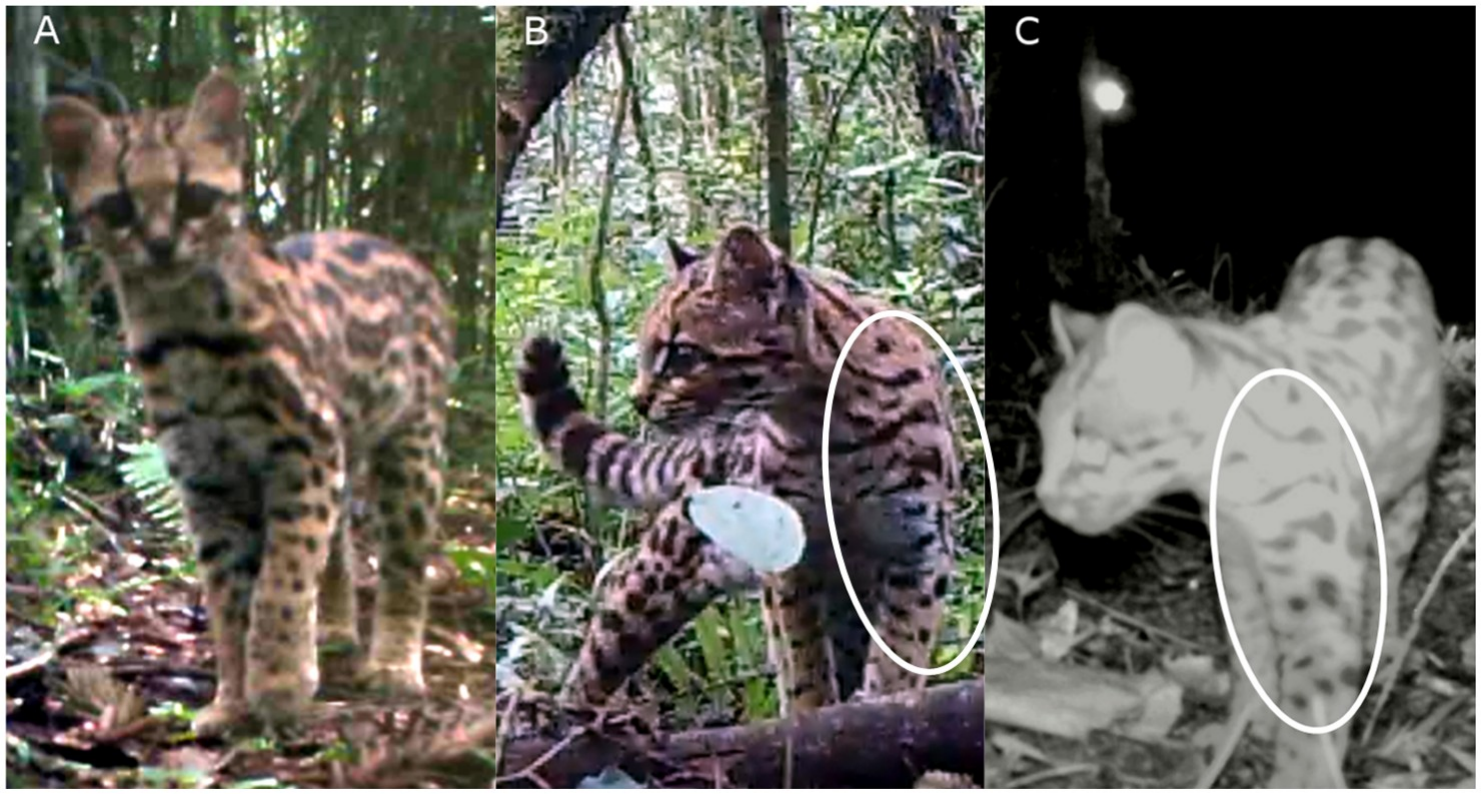
Approach for individual margay identification. A and B are photographs of two different individuals. B and C photographs of the same individual recorded on separate occasions. Individual identification is based on the unique coat pattern on the front right leg and neck (white circle).

We identified individual margays from the videos based on their unique spot patterns (Fig 2) and determined the sex of each individual, whenever possible, through the visualization of presence/absence of male gonads. We used spatial capture-recapture (SCR) models [1,67] following the workflow of package *oSCR* 0.42.0 [66,68] in R 3.6.0 [69] with multi-session sex-structure models to investigate the population density (*D*), the rate of detection (*p*) and the space use (σ) for margay in the study areas.

**Fig 3:**
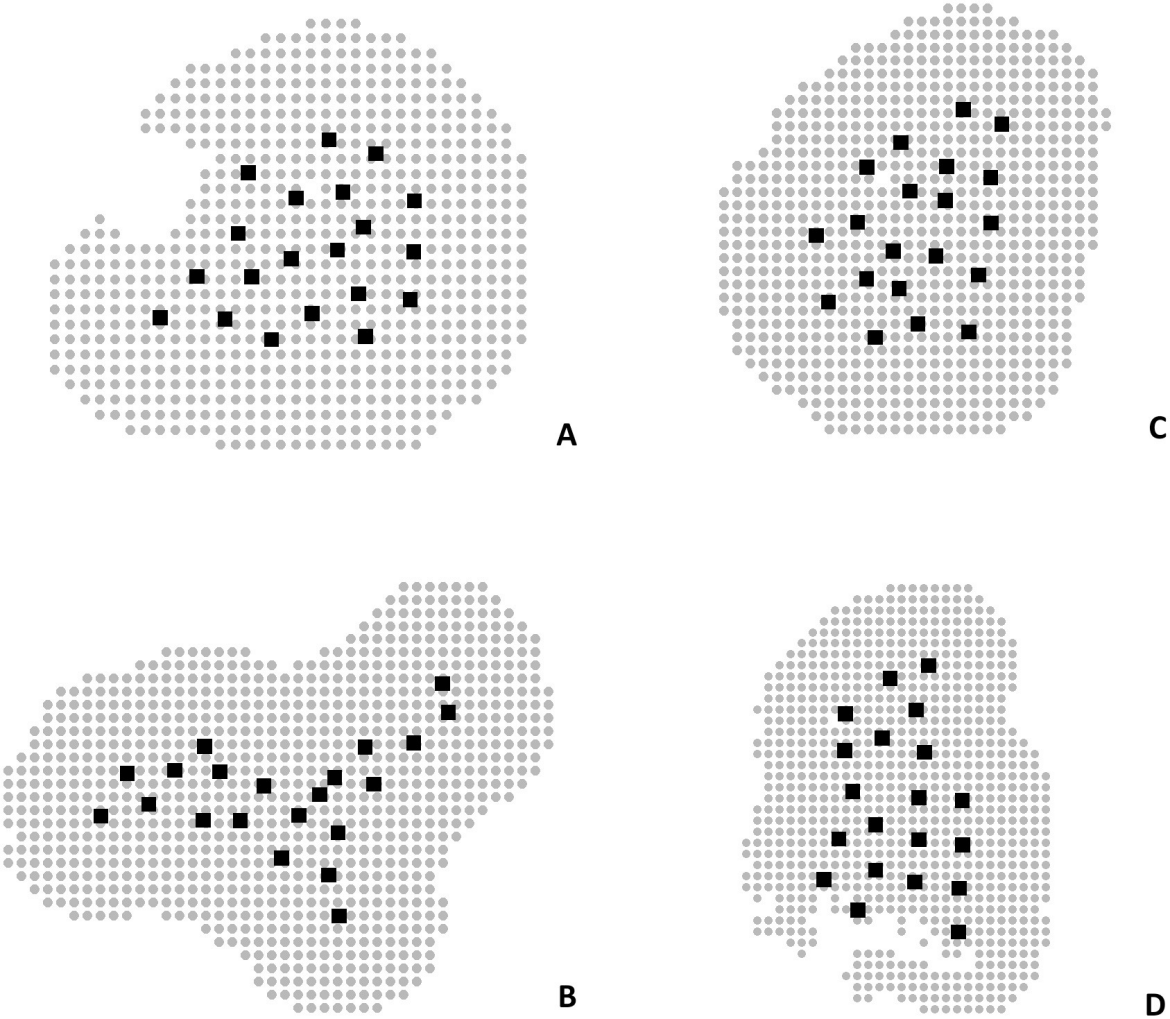
State spaces created for the sampled areas. Black squares represent locations of the camera-trap stations and grey points represent the pixel centroids of the state space of the sampled area. A) Banhado dos Pachecos Wildlife Refuge (BPWR), B) Pró-Mata Center for Research and Conservation of Nature (PROMATA), C) Teutônia (TEUT), D) Passo Fundo National Forest (PFNF). Note the water coordinates removed in A and D.

Each sampling area was considered as harboring a distinct margay population, thus each one represents an independent “session” for the analyses. We removed from the analyses the areas of SGNP and TUSP due the low number of records. To create the *oSCR* data object, a single EDF file (encounter data file) with information on the individual encounter history data for all areas (session, individual ID, trap, occasion, sex) was linked with the TDF files (trap deployment file) of each session. The TDF files contained name and coordinates of each site (camera trap station), the trap-by-occasion binary operation data (1 = operational, 0 = not operational) and trap-specific covariates that either varied with occasion (time varying) or were the fixed site covariates (Table 1). The number of occasions was the same among sessions (60 occasions), except for PFNF (56 occasions). An important component of the SCR analysis is the state space (*S*), that in *oSCR* is required for each session [66]. The state space data object (ssDF—state space data frame) was created by defining a buffer distance around the camera traps and a specific resolution defining the state-space centroids, based on the session-specific trap coordinates. We used a buffer distance of ca. four times the space use parameter (σ) estimated (2000m) and a resolution value of half σ [66], (250 x 250 m) (Fig 3). We clipped out non-habitat points (e.g. water bodies), categorized using the MapBiomas [70] raster, from the buffers to avoid bias in the density estimates [1,71].

We used a three-step approach for modelling margay densities: 1) first we analysed the space used by the individuals, fitting the σ parameter models with covariates, such as the sex (female or male) and the session. In these models we set the *D* and *p* parameters as constants: *D*~1; *p*~1, σ [covariate]; 2) in the second step we investigated potential effects of covariates in the rate of detection (*p*) of margay, including sex, session, and constant or time-varying trap-level covariates [66]. In this step, the σ parameter was set according to the best model resulting from step 1, while the *D* parameter continued to be set as a constant (*D*~1) (D~1, p[covariate], σ[first step]); 3) in this final step we allowed the *D* parameter to vary as a function of a single covariate or of an additive combination of two parameters (D~[covariate], p[second step], σ[first step]). The models built in all steps represent our biological hypotheses regarding the effects of covariates on margay density (*D*), rate of detection (*p*) and spatial scale (σ). We ranked the models using the Akaike Information Criterion (AIC) [72], considering equally fitted models those with ΔAIC ≤ 2 [72]. The covariates presented in the top model or models were considered as possible determinants of species density, rate of detection and spatial use.

### Activity patterns

Activity patterns of the margay were evaluated using date and time of the camera-trap records. To maintain temporal independence between the records and avoid autocorrelation, we only considered those with at least a 1-hour gap [10,73]. We applied the same approach to all other species expected to affect the activity pattern of the margay, including possible prey items (small mammals and small birds), a possible wild competitor (the ocelot), and exotic/domestic species such as domestic dog, cat, cattle and boar.

We tested the uniformity in the activity pattern over the 24 hours of the day for margay, small mammals, and small birds using Rao’s Spacing Test and we applied Watson’s Two-Sample Test of Homogeneity to compare the pairwise distribution of activity of using the package *circular* 0.4–93 [74] in R. If the sample group had homogeneity, we used records of the group from all areas together and applied Rao’s Spacing test to measure the uniformity of activity over 24 hours for the entire region of southernmost Atlantic Forest.

We compared the activity of margay with that of the other species by estimating the coefficient of overlap (Δ) per area. We used the package *overlap* 0.3.2 [75] I n R [76]. The coefficient of overlap (Δ) ranges from 0 (no overlap) to 1 (complete overlap), and is described graphically through a kernel density curve [75,77]. We used the threshold proposed by [7] to classify the degree of overlap between species. We adopted the estimator Δ_1_, for small samples (n>75) [75,77] and performed smoothed bootstraps, with 1000 resamples, to obtain confidence intervals for the Δ_1_ estimator [77].

### Ethics statement

Research permits for all protected areas were obtained from the Brazilian Ministry of the Environment (permits SISBIO-49050 and SISBIO-64647-1) and from the Environment Secretariat of Rio Grande do Sul (permit SEMARS-588), as well as directly from the landowners in the case of private lands. During this study, no animals were captured or handled, and thus no additional permits or protocols were required by the Brazilian law.

## Results

### Field sampling

Our sampling effort resulted in 7220 camera-trap-nights and 66 independent margay records across the six sampling areas; six of these were in BPWR, 27 in PROMATA, 17 in TEUT, 12 in PFFN, two in SGNP and two in TUSP. The two latter sites were thus removed from subsequent statistical analyses due their small sample size.

Also, we obtained 3089 independent records of small birds (Columbidae and Passeriformes), 289 of small mammals (small rodents and small marsupials) and seven of ocelot (*Leopardus pardalis*). In addition we obtained the following exotic and domestic species independent records: 51 of domestic cat (*Felis catus*), 75 of domestic dog (*Canis familiaris*), 29 of wild boar (*Sus scrofa*), 51 of cattle (*Bos taurus*, *Equus caballus*); some of these were only detected in some of the sampled areas (S2 Table).

### Population density estimates

We were able to individually identify 23 margays, in the four areas considered for analysis: two in BPWR, 10 in PROMATA, six in TEUT, and five in PFNF. We identified the sex of 19 individuals and in four individuals we were unable to determine the sex. We discarded from the analyses seven records for which individual identifications were not possible, due the low-quality videos, totalling 55 available records for the density analysis. The individual encounter frequency ranged from six captures of one individual in the same trap, to only a single capture event (for eight of the individuals). The four sampling areas considered for the analyses and the respective state space are shown in Fig 3.

**Fig 4:**
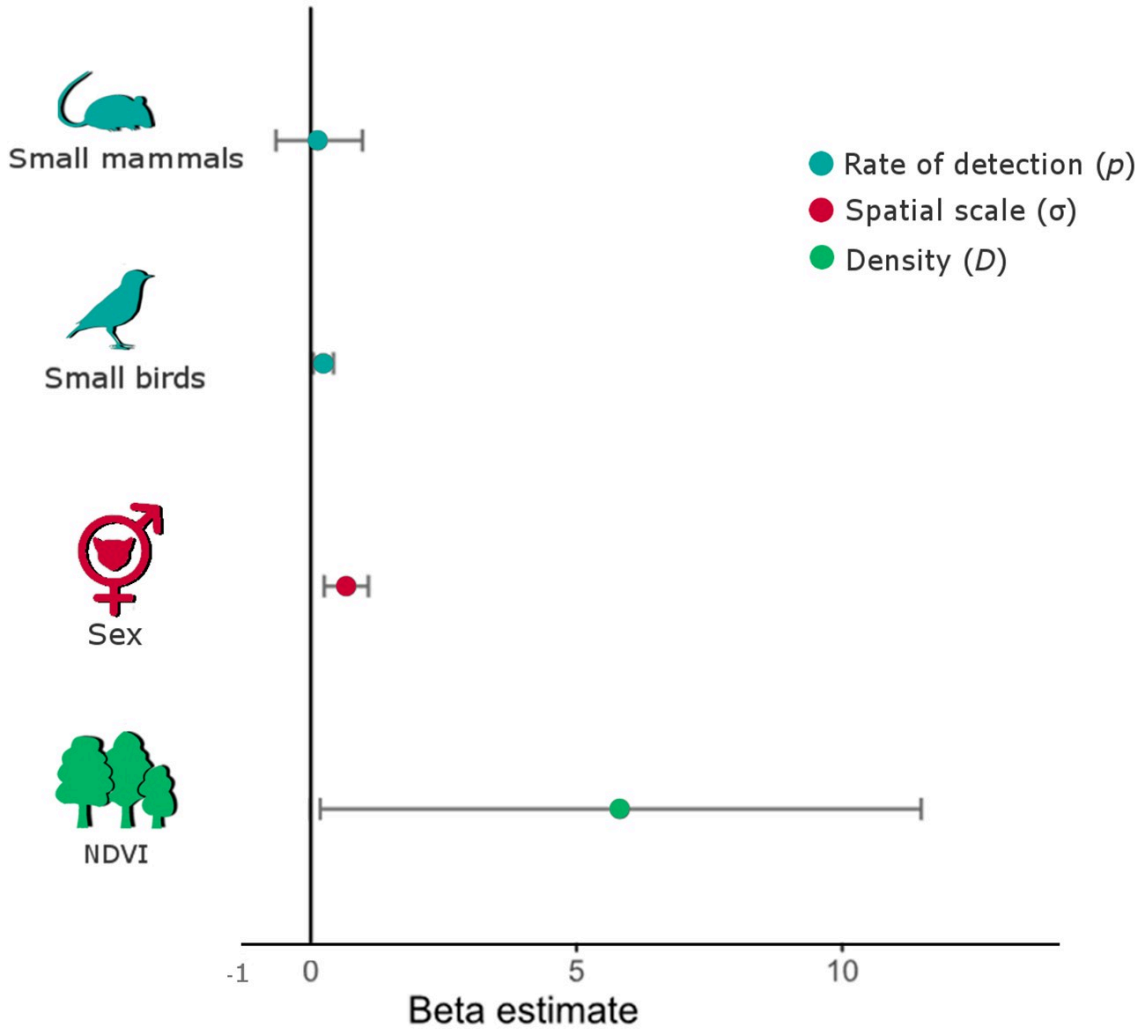
Covariate effect on the density (*D*), spatial scale (σ) and rate of detection (*p*) of margay in the study area. P-values for each of the covariates on the basic parameter based on estimates from the spatial capture–recapture best model: vegetation cover/ndvi (0.050), sex (0.008), behavior (0.001), small birds (0.030), and small mammals (0.770).

For the first step, regarding models created to test covariates on the spatial scale, only one model returned ΔAIC ≤2, representing 79% of the weight of the models (Table 2). In the spatial scale of detection, sex influenced significantly the σ movement parameter (β = 0.66, 0.27–1.06 CI), which was larger for males (1.19 km) than for females (0.59 km) (Fig 4). In the second step, regarding the 13 alternative models testing covariate effect on rate detection, the two-top ranked (ΔAIC <2) represented 54% of the weight of all models (Table 3). The first model estimated a small but positive influence of small bird detection on the rate of detection of the margay (β = 0.23 ± 0.09 SE [0.05–0.41 CI], P = 0.02, weight = 39%), while the second best estimated similar influence of this covariate, and an additional effect of small mammal detection, though not significant (β = 0.12± 0.42 SE [-0.67–0.93 CI], P = 0.794) (Fig 4). Because biologically it makes sense that detections of margay respond to the presence of potential prey, we choose the second ranked model as best fitted, and kept it for the spatial variation density models.

**Table 2:** Candidate set models evaluating the role of covariates on the spatial scale (σ) of the margay (AICc: Akaike Information Criteria for small sample sizes; ΔAICc: Difference between AICc of each model and the model with the lowest AICc; w: Weight).

Density (D)	Detection (p0)	σ	Log-likelihood	N° of parameters	AICc	ΔAICc	w
1	1	sex	358.24	4	726.47	0.00	0.79
1	1	sex+session	357.69	7	729.37	2.90	0.19
1	1	1	363.20	3	734.40	7.93	0.01
1	1	session	360.89	6	735.78	9.31	0.01

**Table 3:** Candidate set models evaluating the role of covariates on rate of detection (p0) of margay (σ: Spatial scale, AICc: Akaike Information Criteria for small sample sizes; ΔAICc: Difference between AICc of each model and the model with the lowest AICc; w: Weight).

Density (D)	Detection (p0)	σ	Log-likelihood	N° of parameters	AICc	ΔAICc	w
1	sbirds	best	355.89	5	723.78	0	0.39
1	sbirds+small	best	355.86	6	725.72	1.93	0.15
1	1	best	358.23	4	726.47	2.68	0.10
1	dogs	best	357.52	5	727.04	3.26	0.07
1	cows	best	357.75	5	727.50	3.71	0.06
1	trigger	best	358.07	5	728.14	4.35	0.04
1	pir	best	358.11	5	728.22	4.43	0.04
1	ocelot	best	358.17	5	728.35	4.57	0.04
1	small	best	358.18	5	728.37	4.59	0.03
1	trigger+pir	best	357.57	6	729.14	5.35	0.02
1	session	best	357.60	7	731.20	7.42	0.00
1	1	1	363.20	3	734.40	10.61	0.00
1	cats	1*	363.04	4	736.09	12.30	0.00

* The model D(.)*p*(cats)σ(sex) did not worked, we then created a model D(.) *p*(cats)σ(.) in order to not exclude the use of “cats” covariable.

**Fig 5:**
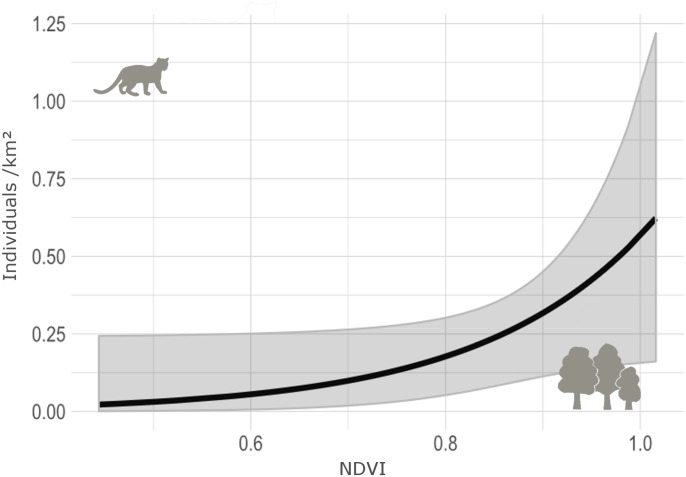
Effect of the vegetation cover (NDVI) on margay’s density estimates (ind/km^2^) in the sampled areas.

**Fig 6:**
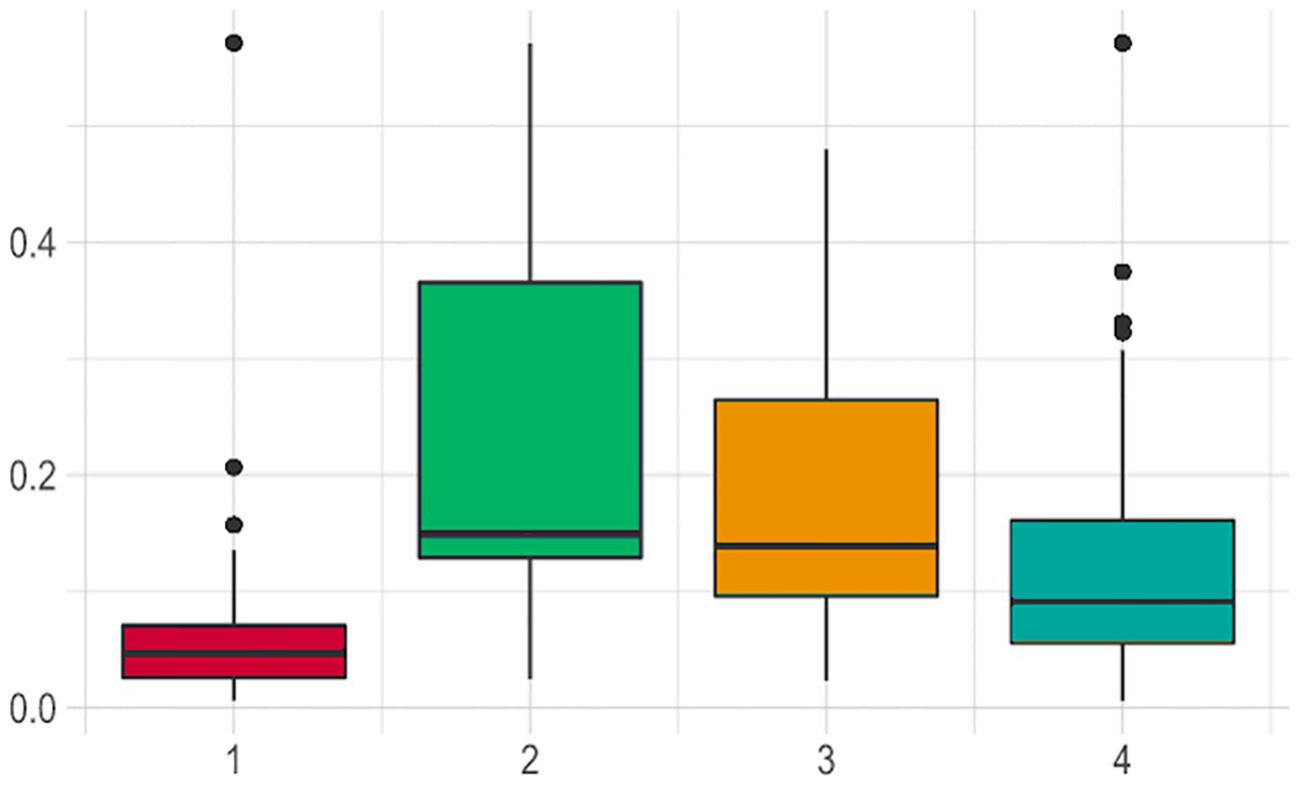
Margay density estimates in the four sampled areas for which estimation was possible (individuals per 100 km^2^). Each session corresponds to a sampling area: 1) Banhado dos Pachecos Wildlife Refuge (BPWR), 2) Pró-Mata Center for Research and Conservation of Nature (PROMATA), 3) Teutônia (TEUT), 4) Passo Fundo National Forest (PFNF). The length of each box shows the range within the upper and lower quartile density estimates values, and the vertical lines shows the estimated maximum and minimum density values. The median is represented by horizontal bar inside each box. The black dots represent outliers.

**Fig 7:**
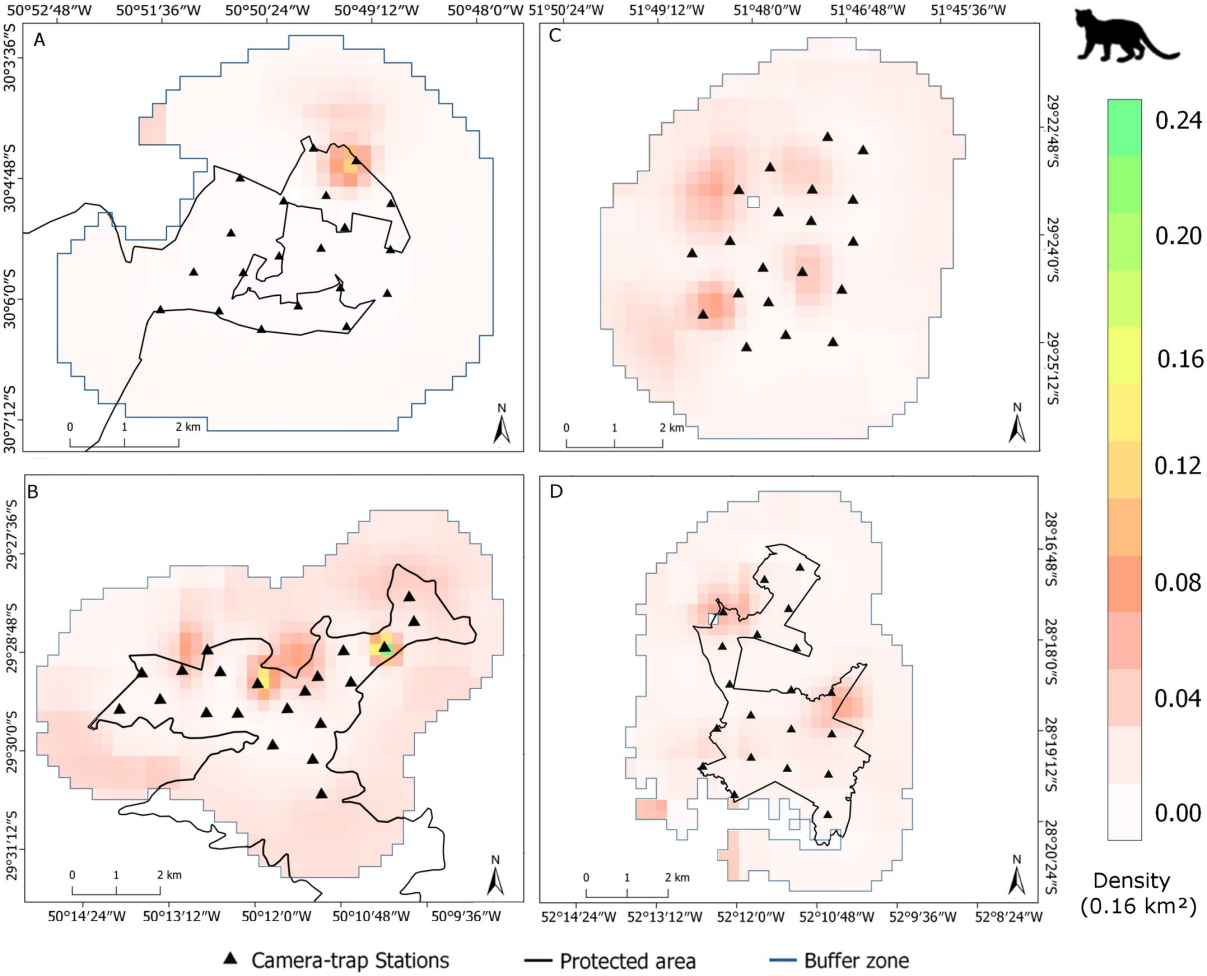
Margay density maps in the sampled areas. Realized density based on estimates of the best SCR model derived from the camera-trap sampling. A) Banhado dos Pachecos Wildlife Refuge (BPWR), B) Center for Research and Conservation of Nature Pró-Mata (PROMATA), C) Teutônia (TEUT), D) Passo Fundo National Forest (PFNF). Water bodies were removed in A and D. Density scales are specific to each map. Pixel resolution at 250 x 250m; density scales are in margay individuals per 0.16 km^2^.

From the nine candidate models in the third modelling step, the top model included only vegetation cover as covariate for the density of the margay, with 33% of the weight of all models (Table 4), with a marginally positive influence on the density (β = 5.81±3.01, SE [0.14–11.4 CI], P = 0.05), (Figs 4 and 5). We disregarded the second and third models, because none of the covariates (distance of water and population density) were statistically significant. Density estimates varied between the studied areas (Fig 6); moreover, the model including the session covariate (comparison between areas) indicated significant differences between the densities in BPWR and PROMATA (P = 0.04); however, this model was not top ranked (Table 4). The density estimates from the top-ranked *oSCR* model varied between the areas: 9.6±6.4 individuals/100km^2^ in BPWR; 37.4±15.1 individuals/100km^2^ in PROMATA; 29.6±11.4 individuals/100km^2^ in TEUT; 28.4±12.5 individuals/100km^2^ in PFNF (Figs 6 and 7).

**Table 4:** Candidate models evaluating the role of covariates on margay density (*D*) (σ: Spatial scale, AICc: Akaike Information Criteria for small sample sizes; ΔAICc: Difference between AICc of each model and the model with the lowest AICc; w: Weight).

Density (D)	Detection (p0)	σ	Log-likelihood	N° of parameters	AICc	ΔAICc	W
ndvi	best	best	353.46	7	712.57	0.00	0.37
ndvi + diswater	best	best	353.36	8	714.56	1.99	0.13
popdens	best	best	354.50	7	714.73	2.16	0.12
diswater	best	best	354.72	7	715.37	2.79	0.09
disroads	best	best	354.72	8	715.67	3.09	0.07
1	best	best	355.86	6	715.69	3.12	0.07
popdens + disroads	best	best	353.96	8	716.14	3.57	0.06
session	best	best	353.14	9	716.84	4.27	0.04
1	1	1	363.20	3	734.40	21.83	0.00

### Activity patterns

**Fig 8:**
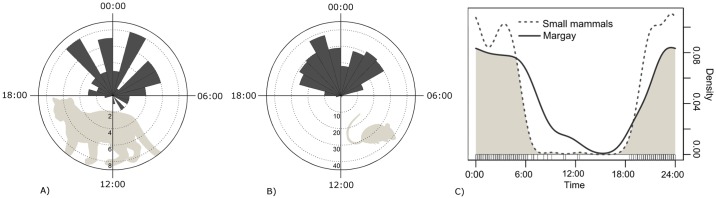
Daily activit y patterns and temporal overlap of margay and small mammals for the sampled region as whole. A) margay, B) small mammals, C) temporal overlap of margay and small mammals.

**Fig 9:**
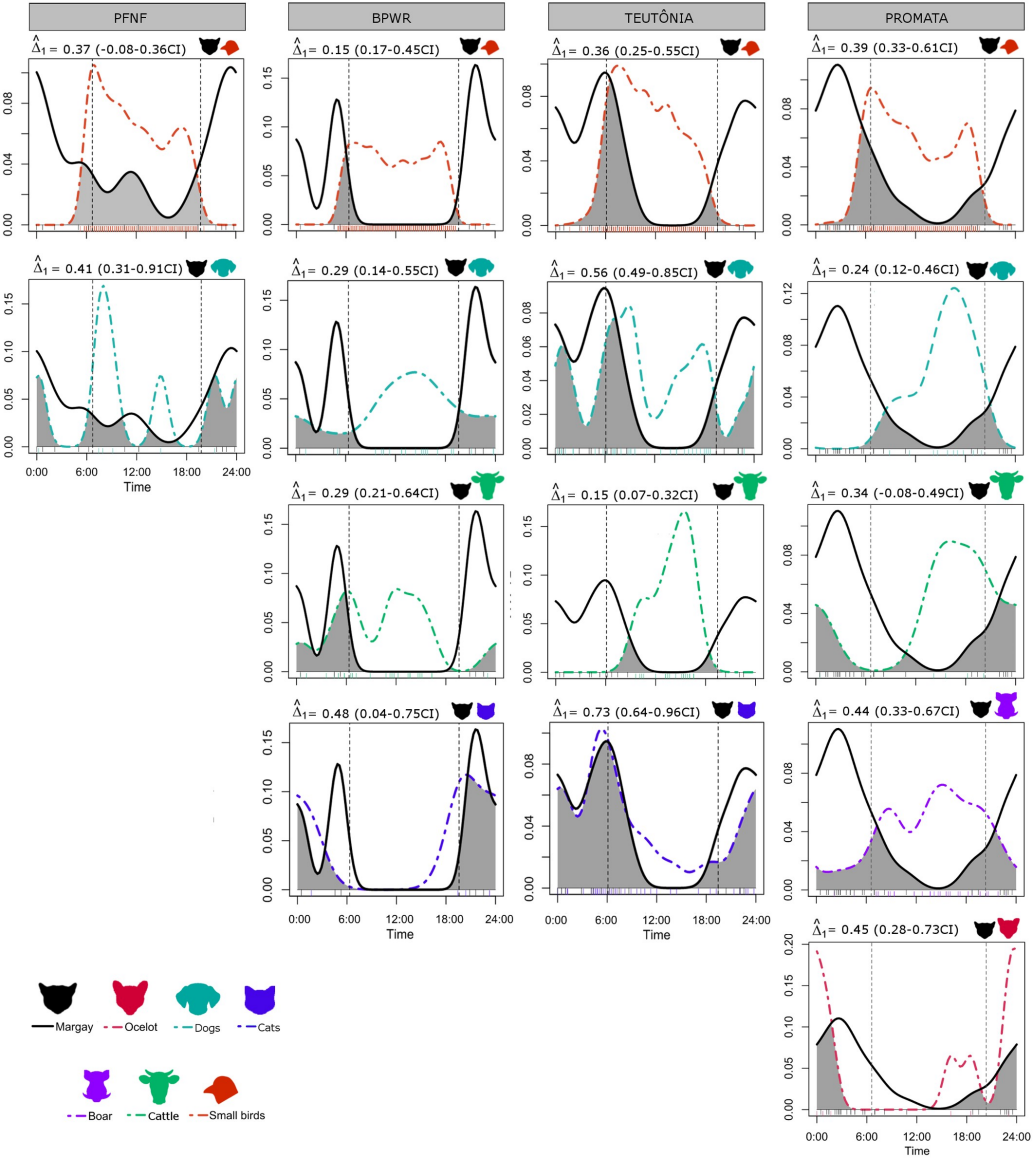
Activity and temporal overlap (Δ_1_, CI) of margay and the remaining evaluated species in the study area—Ocelot, domestic dog and cat, boar, cattle and small birds. Overlap coefficient (Δ_1_) between species is indicated by the shaded area. The dotted grey lines represent sunset and sunrise times during the sampled period in each area (S3 Table).

To evaluate the activity pattern of the margay we considered 62 independent records, excluding the records from SGNP and TUSP (n = 2 for each). Because there were no significant differences in margay and small mammal activity patterns between the sampled areas (Table 5), we merged the records for each taxon to tested for uniformity in the activity pattern over the 24 hours of the day. Margay showed statistically significantly non-uniform activity pattern (Rao’s Spacing Test, r = 261.29; P< 0.001), with average between 01:00 and 02:00am (Fig 8A). Regarding the activity patterns of the potential prey, small mammals also presented non-uniform activity pattern throughout the daily cycle (Rao’s Spacing Test r = 341.31, P< 0.001) with average at 00:00 (Fig 8B). Temporal coefficient overlap between margay and small mammals was 0.73 (0.62–0.87 CI) (Fig 8C). The activity pattern of small birds was dissimilar between the sampled areas (Table 5), so activity overlap between margay and small birds was tested separately for each area; temporal coefficient overlap between margay and small birds ranged from 0.15 to 0.39 (Fig 9).

**Table 5:** Estimates of Watson’s Two-Sample Test of Homogeneity of margay and its potential prey; estimates (P-values).

		Watson’s Two-Sample			
		Test			
		BPWF	PROMATA	TEUT	PFNF
Margay	**BPWF**	.	0.07 (>0.10)	0.06 (>0.10)	0.05 (>0.10)
Small birds			0.69 (<0.00)	0.91 (<0.00)	0.47(<0.00)
Small mammals			0.15 (>0.10)	0.08 (>0.10)	0.08 (>0.10)
Margay	**PROMATA**	.	.	0.10 (>0.10)	0.11 (>0.10)
Small birds				1.11 (<0.00)	0.55 (<0.00)
Small mammals				0.10 (>0.10)	0.17 (>0.10)
Margay	**TEUT**	.	.	.	0.04 (>0.10)
Small birds					0.32 (<0.01)
Small mammals					0.13 (>0.10)
Margay	**PFNF**	.	.	.	.
Small birds					
Small mammals					

Overlap coefficients between margay and dogs ranged from 0.24 to 0.56 (Fig 9). The estimation of the temporal overlap between margay and domestic cats was only possible for BPWR and TEUT, with 0.48 and 0.73 overlap coefficients, respectively (Fig 9). The lowest temporal overlap estimated was with humans, cattle (horse and cow) and small birds (Fig 9). Temporal overlap between margay and ocelot, and margay and wild boar were estimated only for PROMATA, resulting in 0.45 and 0.44 overlap coefficients, respectively (Fig 9).

## Discussion

Few studies estimated the density of margay [30,36,78] and compared temporal activities of any Neotropical small cats with human related species or human related impacts [17,79]. Our study represents the first large-scale evaluation of density and activity patterns of the margay for the southernmost limit of the Atlantic Forest and may be used as model for other studies seeking the management and conservation of small felids.

### Margay: Undoubtedly a forest cat

In the southern limit of the Brazilian Atlantic Forest, the margay seems to occur at higher densities in areas with higher vegetation cover, as hypothesized, probably resulting from the known arboreal habit of the species [20,23]. This arboreal behavior also seems to contribute to the potential relevance of small birds in the diet of margays [40,80]; indeed, the rate of detection of small birds positively impacted rate of detection of the margay. Though we did not evaluate diet composition, this pattern was expected as small birds and small mammals constitute important food resources for the species [40,80]. The effect of the detection of small mammals on margay’s detection was not statistically significant as occurred with the detection of small birds. Indeed, we obtained a low number of small mammal records, which may have resulted from the camera-trap position (angle), as distinct camera-trap position is necessary for the maximum detection of small mammals [81,82].

Margay males seem to walk longer distances (1.19 km) than females (0.59 km). A similar pattern was already reported in Mexican margay populations, who estimated average home ranges of 4.1 km^2^ for males and of 0.72 km^2^ for females [52]. Similar differences have been reported for other felids in the Atlantic Forest, including the jaguar and the ocelot [83,84]. Indeed, larger home ranges of solitary males of the Carnivora seem to respond to resource availability and as response to reproductive opportunities [85]. We found a lower use of space by females than reported by IUCN for the Mexican margay populations [22,52]. Reported influence of roads and human occupation on jaguar female use of space [86] may perhaps explain our results with the margay.

The highest densities of margay occurred in PROMATA, the largest privately protected area of Rio Grande do Sul, and where ocelots seem to be rare (unpublished data; (S2 Table). This area presents low level of human disturbance [59] and is in large part occupied by primary forests, and forests and natural fields under natural regeneration from agricultural use for the last two decades [87]. The existence of high margay densities indicates that, unless a landscape is profoundly modified, active management practices towards forest regeneration should allow the persistence of a significant number of individuals in smaller fragments, most likely because arboreal shelters and prey exist in sufficient numbers to reduce intraspecific competition [88,89].

Consistently, the lowest density estimate was obtained for BPWR. In this area, forest coverage is small, as the area is dominated by open physiognomies such as permanent wetlands, marshlands, restingas and dry grasslands [90] and, indeed, suitability models suggest the species to be negatively correlated to flooded grasslands and savannas [24]. From our sampled areas, the BPWR is that in the southernmost latitude, and situated in the ecotone between the Atlantic Forest and the Brazilian Pampa. In Pampean landscapes, the marginal occurrence of the margay should be tied with the natural forested fragments of the biome, occurring mostly bordering water courses [24]. Besides that, the BPWP, although a strictly protected conservation unit, is located only 28 km away from the largest urban center of Rio Grande do Sul (Porto Alegre, with ca.1,409 million habitants) [59], inevitably suffering from the expected associated impacts: illegal hunting, water drainage, and intensive agricultural schemes, particularly rice production [91].

TEUT and PFNF presented intermediate densities suggesting similar responses of margay populations in these two areas. PFNF is a relevant representative of the Araucaria Forest physiognomy in the southern Atlantic Forest, showing a gradient of regeneration after somewhat intensive human use in the last decades [57,58]. Although a conservation area of sustainable use, it is located in a matrix of monocultures and livestock production, representing an isolated fragment with some, while low, connectivity with other forest fragments; agriculture and illegal hunting represent the major negative impacts on the native fauna. These characteristics may explain why PFNF, being a protected area with somewhat similar features to PROMATA, presents densities of margay more similar to TEUT an altogether non-protected area. TEUT is basically an area of small properties with different agricultural uses, harboring forested fragments in those areas considered unsuitable for crop production. The estimated margay density here is higher than that estimated for other neotropical cats, including the southern tiger cat (*Leopardus guttulus*) and the jaguarundi (*Herpailurus yagouaroundi)* (8 individuals/100km^2^ and 4 individuals/100km^2^, respectively [92], though these studies used different methods for estimating population densities).

We were unable to estimate densities for TUSP and SGNP, due to the small number of records obtained for these areas. In TUSP, low margay detection contrasts with high ocelot densities (14 (14–24 CI)– 66 (49–182 CI) individuals/100km^2^ [93] and 15.5 (6.4–36 CI) individuals/100km^2^ [94]). The suspected ‘ocelot effect’ may be affecting margay; still, because this is quite a pristine area, with high native tree cover, we expected to collect enough data to at least estimate the density in the area, which was not the case. In SGNP, we suspect that the somewhat intensive presence of exotic/domestic fauna within the park (cattle, boars and buffalos), as several areas are still ongoing private expropriation, could explain the low number of margay records [24]. Additionally, the area is a mosaic of forest and open fields and, within our sampled sites, is that with the largest proportion of grassland habitats, which may explain the scarce number of records of the margay, positively influenced by vegetation cover and seemingly forest-dependent.

To our knowledge there is no published information on density patterns of the margay in the Atlantic Forest. Our estimates are, however, lower than those reported for southwest and southeastern Mexico, which varied from 12 individuals/100 km^2^ [30] to 81 individuals/100 km^2^ [31], respectively. Overall, margay population densities seem to respond positively to vegetation cover. Higher estimated values of density were found in more preserved areas and the lower values in human-altered landscapes [59]. The intermediate values of density were found in areas with moderate human land use and reduced natural vegetation cover [59], suggesting that conservation strategies focusing on less pristine or small forest fragments may have positive effects on the density of margays across its distribution range.

### Margays prefer the night: Avoiding antagonists or simply following prey?

Margay was nocturnal across our studied areas, consistent with what has been found in other studies [17,30,31,39,95]. In fact, our results did not support our prediction of changes in the activity pattern of the margay in response to human disturbance: margay was strictly nocturnal across a range of human-altered landscapes. This pattern probably co-evolved as a response to the activity pattern of their preferential prey—small mammals [80]—but may have also been intensified in the last centuries, by human disturbance [15], including hunting pressure and the presence of antagonists—potential predators such as domestic dogs or simply antagonists in the sense of profound habitat modifiers such as wild boars [79,96,97].

Small mammals are recognized as the most important dietary item of margays [40,98,99]. Similar nocturnal activity patterns probably co-evolved over millions of years increasing on the one hand the chances of prey capture by the margay, but on the other, increasing possibilities of the potential prey to escape. However, small birds, also an important item in diet of margay [40,80], are mostly diurnal, suggesting that if eaten in our sampled areas, they are probably preyed upon while resting [100].

Ocelot activity did not overlap significantly with that of the margay, and that is a pattern already described for southeast remnants of the Brazilian Atlantic Forest [40]. Temporal segregation facilitates species coexistence, by reducing competition for space and prey [6,40,101]. However, it is important to notice that the number of ocelot records in our study was small, and more data will be necessary to support this conclusion. Apart from temporal segregation, segregation by vertical stratification between the ocelot and the margay still needs to be tested, as this has been suggested as the factor behind the higher activity overlap between the two species in the Argentine Atlantic Forest [17]. Moreover, the margay nocturnal activity reported in this study can be associated with a temporal segregation between this species and another felid, as diurnal jaguarundi (*Herpailurus yagouaroundi*) [6,17]. These two species seem to have the highest similarity in morphological traits associated with trophic ecology among Neotropical cats, and the contrasting activity pattern may be relevant to allow their coexistence [6,102].

Temporal overlap between the margay and domestic dogs was low in general, with the highest overlap occurring in TEUT. Dogs presented diurnal activity, consistently with the pattern found in other Atlantic Forest areas [103,104]. Theoretically, such low overlap suggests that domestic dogs probably do not prey upon margays. Still, they may have negative indirect effects, representing an important cause of decreased prey populations [105] or as reservoirs for pathogenic agents [106]. Domestic cats, on the other hand, revealed to be mostly nocturnal, and this was particularly evident in TEUT where a considerable higher number of domestic cat records was obtained, probably related to the higher number of human habitations [107]. Domestic cats, therefore, may represent a potentially important competitor for food regarding the margay and other felids (e.g. southern tiger cat, jaguarondi), as they show similar opportunistic predatory behavior [97,108]. Disease transmission is a very likely indirect negative impact resulting from the occurrence of both species in the wild, representing an additional threat to native felids [18,107–111].

We also found cattle and wild boar in some of the studied areas, although both were mainly diurnal. Potential impacts of both species on margay populations are thus probably related to changes in the landscape [112–116], especially by the wild boar, known for intense nest predation and destruction of native wildlife habitats [96,117–119].

## Conclusion

To our knowledge, this is the first study specifically attempting to estimate multi-area density and activity patterns of the margay, and also to compare the activity pattern of the margay with those of human related species. Our results supported our hypothesis that densities differ across the sampled areas, reflecting differences in the composition of the landscape and in the levels of human disturbance. On the other hand, we found no significant changes in the activity pattern of the species between the sampled areas. Indeed, the margay seems to be mostly a forest nocturnal cat, whose densities are positively influenced by forest cover and negatively influenced by intense human-related disturbances, while not changing its activity pattern across landscapes with distinct intensities of human use. Undoubtedly, large pristine forest areas, with high prey—small birds and mammals—availability, are critical for the persistence of dense populations of the species. However, under moderate levels of habitat modification and human disturbance, the margay is still able to persist—at least at intermediate densities—suggesting that the conservation of even small native forest remnants, especially those showing some degree of connectivity among them, is key for margay population management and conservation at the southern limit of the Brazilian Atlantic Forest.

## Supporting information

S1 Table Variance inflation factor test results for the density covariates models. (PDF)

S2 Table Total and independent records for small mammals, small birds, ocelot, domestic cat, domestic dog, wild boar, cattle, and humans. (PDF)

S3 Table Mean and standard deviation for time of sunrise and time of sunset of the study period in each area; data obtained for Viamão, São Francisco de Paula, Teutônia and Passo Fundo municipalities (which include our study areas), RS, Brazil. (PDF)

S1 Appendix Margay records used in data analysis. Area, time, coordinates, trap names, occasion and ID of each margay record used in data analysis.
(CSV)

S2 Appendix Trap Deployment File (TDF) from the areas. Names, coordinates, operation data and specific covariates of each trap.
(CSV)

S3 Appendix Encounter data file from margay in the areas. Individual encounter history data with information of trap names, occasions, sessions and sex in eaxh area where margays were detected.
(CSV)

S4 Appendix State space for each session. (CSV)

S5 Appendix Predicted values from the fitted density model. Density values in each pixel of the state space, containing a column with the spatial covariate used in the model component (vegetation cover).
(CSV)

S6 Appendix Produced predicted values from sex-specific sigma. (CSV)

S7 Appendix Beta estimate values from fitted density model covariates. (CSV)

S8 Appendix Analysis data in the R package “circular”. (CSV)

S9 Appendix Analysis data in the R package “overlap”. (CSV)

S10 Appendix R script text file. Code run to perform the analysis of density and activity patterns of margay.
(TXT)

## References

[1] Royle JA, Chandler RB, Sollmann R, Gardner B. Spatial Capture-recapture: First Edition First Edit. Spatial Capture-recapture. Waltham: Academic Press; 2014 1–577 p.

[2] IUCN Standards and Petitions Subcommittee. Guidelines for Using the IUCN Red List Categories and Criteria. Vol. Version 13. 2017.

[3] Paviolo A, De Angelo CD, Di Blanco YE, Di Bitetti MS. Jaguar Panthera onca population decline in the Upper Paraná Atlantic Forest of Argentina and Brazil. Oryx. 2008;42(4):554–61.

[4] Strier KB. Population Viabilities and Conservation Implications for Muriquis. Popul (English Ed. 2000;32(April 1999):903–91.

[5] Bernardo CSS, Rubim P, Bueno RS, Begotti RA, Meirelles F, Donatti CI, et al Density Estimates of the Black-Fronted Piping Guan in the Brazilian Atlantic Rainforest. Wilson J Ornithol. 2011;123(4):690–8.

[6] Di Bitetti MS, De Angelo CD, Di Blanco YE, Paviolo A. Niche partitioning and species coexistence in a Neotropical felid assemblage. Acta Oecologica. 2010;36(4):403–12.

[7] Monterroso P, Alves PC, Ferreras P. Plasticity in circadian activity patterns of mesocarnivores in Southwestern Europe: Implications for species coexistence. Behav Ecol Sociobiol. 2014;68(9):1403–17.

[8] Gómez-Ortiz Y, Monroy-Vilchis O, Castro-Arellano I. Temporal coexistence in a carnivore assemblage from central Mexico: temporal-domain dependence. Mammal Res. 2019;64(3):333–42.

[9] Harmsen BJ, Foster RJ, Silver SC, Ostro LET, Doncaster CP. Spatial and Temporal Interactions of Sympatric Jaguars (Panthera onca) and Pumas (Puma concolor) in a Neotropical Forest. J Mammal. 2009;90(3):612–20.

[10] Foster VC, Jácomo ATA, Tôrres N, Fonseca C, Negrões N, Sollmann R, et al Jaguar and Puma Activity Patterns and Predator-Prey Interactions in Four Brazilian Biomes. Biotropica. 2013;45(3):373–9.

[11] Hofmann GS, Coelho IP, Bastazini VAG, Cordeiro JLP, de Oliveira LFB. Implications of climatic seasonality on activity patterns and resource use by sympatric peccaries in northern Pantanal. Int J Biometeorol. 2016;60(3):421–33. 10.1007/s00484-015-1040-8 26219606

[12] Wolfe JL, Tan Summerlin C. The influence of lunar light on nocturnal activity of the old-field mouse. Anim Behav. 1989;37(PART 3):410–4.

[13] Lucherini M, Reppucci JI, Walker RS, Villalba ML, Wurstten A, Gallardo G, et al Activity Pattern Segregation of Carnivores in the High Andes. J Mammal. 2009;90(6):1404–9.

[14] Rich C, Longcore T. Ecological Consequences of Artificial Night Lighting. Rich C, Longcore T, editors. London: Island Press; 2006 480 p.

[15] Gaynor KM, Hojnowski CE, Carter NH, Brashares JS. The influence of human disturbance on wildlife nocturnality. Science (80-). 2018;360:1232–5. 10.1126/science.aar7121 29903973

[16] Carter NH, Shrestha BK, Karki JB, Pradhan NMB, Liu J. Coexistence between wildlife and humans at fine spatial scales. Proc Natl Acad Sci U S A. 2012;109(38):15360–5. 10.1073/pnas.1210490109 22949642 PMC3458348

[17] Cruz P, Iezzi ME, De Angelo C, Varela D, Di Bitetti MS, Paviolo A. Effects of human impacts on habitat use, activity patterns and ecological relationships among medium and small felids of the Atlantic Forest. PLoS One. 2018;13(8):1–21. 10.1371/journal.pone.0200806 PMC6070200 30067785

[18] Macdonald DW, Loveridge AJ. Biology and Conservation of Wild Felids. New York: Oxford University Press Inc; 2010 1–762 p.

[19] de Oliveira TG, Cassaro K. Guia de identificação dos felinos Brasileiros. 1997 1–60 p.

[20] Sunquist F, Sunquist M. The Wild Cat Book: Everything you ever wanted to know about cats. Chicago: University Of Chicago Press; 2014 1–280 p.

[21] Hunter L. Wild Cats of the World. 1st ed London: Bloomsbury Natural History; 2015 240 p.

[22] de Oliveira T, Paviolo A, Schipper J, Bianchi R, Payan E, Carvajal SV. Leopardus wiedii. The IUCN Red List of Threatened Species 2015. 2015. p. 1.

[23] Sunquist M, Sunquist F. Wild Cats of the World. Chicago: University of Chicago Press; 2002 1–462 p.

[24] Espinosa CC, Trigo TC, Tirelli FP, da Silva LG, Eizirik E, Queirolo D, et al Geographic distribution modeling of the margay (Leopardus wiedii) and jaguarundi (Puma yagouaroundi): a comparative assessment. J Mammal. 2018;99(1):252–262.

[25] de Oliveira TG. Leopardus wiedii. Mamm Species. 1998;579:1–6.

[26] Myers N, Mittermeier RA, Mittermeier CG., da Fonseca GAB, Kent J. Biodiversity hotspots for conservation priorities. Nature. 2000;403(6772):853–8. 10.1038/35002501 10706275

[27] Ribeiro MC, Metzger JP, Martensen AC, Ponzoni FJ, Hirota MM. The Brazilian Atlantic Forest: How much is left, and how is the remaining forest distributed? Implications for conservation. Biol Conserv. 2009;142(6):1141–53.

[28] Brown JH. On the relationship between abundance and distribution of species. Am Nat. 1984;124:255–79.

[29] Gaston KJ. Patterns in the geographical ranges of species. Biol Rev. 1990;65:105–29.

[30] López-Hernández LD. Abundancia y patrón de actividad de Leopardus wiedii en la Sierra Nanchititla, México. Tesis de Licenciatura. Universidad Autónoma del Estado de México; 2010.

[31] Pérez-Irineo G, Santos-Moreno A, Hernández-Sánchez A. Density and activity pattern of Leopardus wiedii and Leopardus pardalis in Sierra Norte of Oaxaca, Mexico. Therya. 2017;8(3):223–32.

[32] De Oliveira TG, Tortato M a, Silveira L, Kasper CB, Mazim FD, Lucherini M, et al Ocelot ecology and its effect on the small-felid guild in the lowland neotropics. Biol Conserv wild felids. 2010;(July):559–80.

[33] Oliveira TG De, Mazim FD, Fox-rosales L, Peters FB, Rosane V, Lima BC, et al. Assessing Small Cats Abundance in Brazil: Camera Trapping Summary Report– 2018. 2018.

[34] Borchers DL, Efford MG. Spatially explicit maximum likelihood methods for capture-recapture studies. Biometrics. 2008;64(2):377–85. 10.1111/j.1541-0420.2007.00927.x 17970815

[35] Gardner B, Sollmann R, Kumar NS, Jathanna D, Karanth KU. State space and movement specification in open population spatial capture–recapture models. Ecol Evol. 2018;8(20):10336–44. 10.1002/ece3.4509 30397470 PMC6206188

[36] Pérez-Irineo G, Santos-Moreno A. Abundance and activity patterns of medium-sized felids (Felidae, Carnivora) In Southeastern Mexico. Southwest Nat. 2016;61(1):33–9.

[37] Briones-Salas M, Lira-Torres I, Carrera-Treviño R, Sánchez-Rojas G. Relative abundance and activity patterns of wild felids in Chimalapas rainforest, Oaxaca, Mexico. Therya. 2016;7(1):123–34.

[38] Vanderhoff E, Hodge A, Arbogast B, Nilsson J, Knowles T. Abudance and activity patterns of the margay (Leopardus wiedii) at a mid-elevation site in the eastern andes of Ecuador. Mastozoologia. 2011;18(2):271–9.

[39] Oliveira-Santos LGR, Graipel ME, Tortato MA, Zucco CA, Cáceres NC, Goulart FVB. Abundance changes and activity flexibility of the oncilla, Leopardus tigrinus (Carnivora: Felidae), appear to reflect avoidance of conflict. Zool. 2012;29(2):115–20.

[40] Nagy-Reis MB, Iwakami VHS, Estevo CA, Setz EZF. Temporal and dietary segregation in a neotropical small-felid assemblage and its relation to prey activity. Mamm Biol. 2019;95:1–8.

[41] Instituto Chico Mendes de Conservação da Biodiversidade ICMBio, Centro Nacional de Pesquisa e Conservação de Mamíferos Carnívoros CENAP. Plano de Açao Nacional para a Conservação dos Pequenos Felinos. 2018. p. 10.

[42] Massara RL, Paschoal AM de O, Bailey LL, Doherty PF, Barreto M de F, Chiarello AG. Effect of humans and pumas on the temporal activity of ocelots in protected areas of Atlantic Forest. Mamm Biol. 2018;92(May):86–93.

[43] Tortato MA, Oliveira TG, Almeida LB, Beisiegel BM. Avaliação do risco de extinção do Gato-maracajá Leopardus wiedii (Schinz, 1821) no Brasil. Biodiversidade Bras. 2013;3(1):76–83.

[44] Estado do Rio Grande do Sul. Decreto No 51.797, 8 de setembro de 2014. Declara as espécies da fauna silvestre ameaçadas de extinção no estado do Rio Grande do Sul. Governo do Estado do Rio Grande do Sul. 2014.

[45] Oliveira-Filho AT, Fontes MAL. Patterns of Floristic Differentiation among Atlantic Forests in Southeastern Brazil and the Influence of Climate1. Biotropica. 2000;32(4):793–810.

[46] Silva LCR, Anand M. Mechanisms of Araucaria (Atlantic) Forest Expansion into Southern Brazilian Grasslands. Ecosystems. 2011;14(8):1354–71.

[47] Morellato LPC, Haddad CFB. Introduction: The Brazilian Atlantic Forest1. Biotropica. 2006;32(4):786.

[48] Hansen AJ, di Castri F. Landscape boundaries: Consequences for Biotic Diversity and Ecological Flows. Billings WD, Lange OL, Remmert H, editors. Ecol Stud. 1992;92.

[49] Roesch LFW, Vieira FCB, Pereira VA, Schünemann AL, Teixeira IF, Senna AJT, et al The Brazilian Pampa: A fragile biome. Diversity. 2009;1(2):182–98.

[50] Löbler CA, Sccoti AAV, Werlang MK. Contribution to the delineation of Pampa and Atlantic Forest biomes in Santa Maria, RS. Rev eletrônica em Gestão, Educ e Tecnol Ambient. 2015;19(2):1250–7.

[51] de FM dos Santos M, Pellanda M, Tomazzoni AC, Hasenack H, Hartz SM. Mamíferos carnívoros e sua relação com a diversidade de hábitats no Parque Nacional dos Aparados da Serra, sul do Brasil. Iheringia Série Zool. 2004;94(3):235–45.

[52] Carvajal-Villarreal S, Caso A, Downey P, Moreno A, Tewes ME, Grassman LI. Spatial patterns of the margay (Leopardus wiedii; Felidae, Carnivora) at “El Cielo” Biosphere Reserve, Tamaulipas, Mexico. Mammalia. 2012;76(3):237–44.

[53] Mohr LR da S, Périco E, Fonseca VS da S, Mohr AR. The breeding biology, nest success, habitat and behavior of the endangered saffron-cowled blackbird, Xanthopsar flavus (Aves: Icteridae), at an Important bird area (IBA) in rio grande do sul, Brazil. Zoologia. 2017;34:1–10.

[54] BRASIL. Portaria no 443, de 3 de Setembro de 2019.Criação da Reserva Particular do Patrimônio Natural—RPPN Pró-Mata / PUCRS. Brasília,DF: Diário Oficial da União: seção 1; 2019. p. 57–9.

[55] Marques RV, Cademartori CV, Pacheco SM. Mastofauna no Planalto das Araucárias, Rio Grande do Sul, Brasil. Rev Bras Biociências. 2011;9(3):278–88.

[56] Kasper CB, Mazim FD, Soares JBG, De Oliveira TG, Fabián ME. Composição e abundância relativa dos mamíferos de médio e grande porte no Parque Estadual do Turvo, Rio Grande do Sul, Brasil Carlos. Rev Bras Zool. 2007;24(4):1087–100.

[57] Nunes de Sá D, Gerhardt M. Uma história ambiental da Floresta Nacional de Passo Fundo: a aquisição das terras. Rev Int Interdiscip INTERthesis. 2017;13(3):182.

[58] Instituto Chico Mendes de Conservação da Biodiversidade. Plano de Manejo da Floresta Nacional de Passo Fundo. Vol. II. 2011.

[59] Graves VM. The impact of anthropogenic factors on occupancy and abundance of carnivorans at the southern limit of the Brazilian Atlantic Forest. Universidade Federal do Rio Grande do Sul; 2019.

[60] Balme GA, Hunter LTB, Slotow R. Evaluating Methods for Counting Cryptic Carnivores. J Wildl Manage. 2009;73(3):433–41.

[61] Di Bitetti MS, Paviolo A, De Angelo C. Density, habitat use and activity patterns of ocelots (Leopardus pardalis) in the Atlantic Forest of Misiones, Argentina. J Zool. 2006;270(1):153–63.

[62] Naimi B, Araújo MB. Sdm: A reproducible and extensible R platform for species distribution modelling. Ecography (Cop). 2016;39(4):368–75.

[63] LP-DAAC -NASA EOSDIS Land Processes Distributed Active Archive Center. MOD13A2v0006 : MODIS / Terra Vegetation Indices 16-Day L3 Global 1 km SIN Grid. Sioux Falls, South Dakota; 2018.

[64] Fundação Estadual de Proteção Ambiental Henrique Luiz Roessler—FEPAM. Biblioteca Digital FEPAM(Internet). 2019 (cited 2019 Jul 21). http://www.fepam.rs.gov.br/biblioteca/geo/bases_geo.asp

[65] Center for International Earth Science Information Network—CIESIN—Columbia University. Gridded Population of the World, Version 4 (GPWv4): Population Density, Revision 11. Palisades, NY: NASA Socioeconomic Data and Applications Center (SEDAC); 2018.

[66] Sutherland C, Royle JA, Linden DW. oSCR: A Spatial Capture‐Recapture R Package for Inference about Spatial Ecological Processes. Ecography (Cop). 2019;ecog.04551.

[67] Efford MG, Fewster RM. Estimating population size by spatially explicit capture-recapture. Oikos. 2013;122(6):918–28.

[68] Molina S, Fuller AK, Morin DJ, Royle JA. Use of spatial capture–recapture to estimate density of Andean bears in northern Ecuador. Ursus. 2017;28(1):117–26.

[69] Team RC. R: A language and environment for statistical computing. Vienna, Austria: R Foundation for Statistical Computing; 2019.

[70] MapBiomas. Projeto MapBiomas—Coleção v. 3.1 da Série Anual de Mapas de Cobertura e Uso de Solo do Brasil(Internet). 2019 (cited 2019 Jul 21). http://mapbiomas.org/

[71] Royle JA, Magoun AJ, Gardner B, Valkenburg P, Lowell RE. Density estimation in a wolverine population using spatial capture-recapture models. J Wildl Manage. 2011;75(3):604–11.

[72] Burnham KP, Anderson DR. Model Selection and Multimodel Inference: A Practical Information-Theoretic Approach 2nd end Vol. 7, Istech. New York: Springer; 2002 515 p.

[73] Porfirio G, Foster VC, Fonseca C, Sarmento P. Activity patterns of ocelots and their potential prey in the Brazilian Pantanal. Mamm Biol. 2016;81(5):511–7.

[74] Agostinelli C, Lund U. R package “circular”: Circular Statistics (version 0.4–93). 2017.

[75] Ridout MS, Linkie M. Estimating overlap of daily activity patterns from camera trap data. J Agric Biol Environ Stat. 2009;14(3):322–37.

[76] Meredith AM, Ridout M, Meredith MM. Estimates of Coefficient of Overlapping for Animal Activity Patterns. 2018;20.

[77] Meredith M, Ridout MS. Overview of the overlap package. R project. 2018 p. 1–9.

[78] Pérez-Irineo G, Santos-Moreno A, Hernández-Sánchez A. Density and activity pattern of Leopardus wiedii and Leopardus pardalis in Sierra Norte of Oaxaca, Mexico. Therya. 2017;8(3):223–32.

[79] Carvalho WD, Rosalino LM, Godoy MSM, Giorgete MF, Adania CH, Esbérard CEL. Temporal activity of rural free-ranging dogs: implications for the predator and prey species in the Brazilian Atlantic Forest. NeoBiota. 2019;45:55–74.

[80] Bianchi R de C, Rosa AF, Gatti A, Mendes SL. Diet of margay, Leopardus wiedii, and jaguarundi, Puma yagouaroundi, (Carnivora: Felidae) in Atlantic Rainforest, Brazil. Zool. 2011;28(1):127–32.

[81] Villette P, Krebs CJ, Jung TS, Boonstra R. Can camera trapping provide accurate estimates of small mammal (Myodes rutilus and Peromyscus maniculatus) density in the boreal forest? J Mammal. 2016;97(1):32–40.

[82] Dundas SJ, Ruthrof KX, Hardy GESJ, Fleming PA. Pits or pictures: A comparative study of camera traps and pitfall trapping to survey small mammals and reptiles. Wildl Res. 2019;46(2):104–13.

[83] Morato RG, Stabach JA, Fleming CH, Calabrese JM, De Paula RC, Ferraz KMPM, et al Space use and movement of a neotropical top predator: The endangered jaguar. PLoS One. 2016;11(12):1–17. 10.1371/journal.pone.0168176 PMC5193337 28030568

[84] Goulart F, Graipel ME, Tortato M, Ghizoni-Jr I, Oliveira-Santos LG, Cáceres N. Ecology of the ocelot (Leopardus pardalis) in the Atlantic Forest of Southern Brazil. Neotrop Biol Conserv. 2009;4(3):137–43.

[85] Sandell M. The Mating Tactics and Spacing Patterns of Solitary Carnivores In: JL G, editor. Carnivore Behavior, Ecology, and Evolution. 1st ed New York: Cornell University Press; 1989 p. 164–82.

[86] Colchero F, Conde DA, Manterola C, Chávez C, Rivera A, Ceballos G. Jaguars on the move: Modeling movement to mitigate fragmentation from road expansion in the Mayan Forest. Anim Conserv. 2011;14(2):158–66.

[87] Moura L de A (ed.. Plano de manjeo—Centro de Pesquisas e Conservação da natureza Pró-Mata. 2011.

[88] Cunningham SC, Mac Nally R, Baker PJ, Cavagnaro TR, Beringer J, Thomson JR, et al Balancing the environmental benefits of reforestation in agricultural regions. Perspect Plant Ecol Evol Syst. 2015;17(4):301–17.

[89] Ramírez-Mejía AF, Sánchez F. Activity patterns and habitat use of mammals in an Andean forest and a Eucalyptus reforestation in Colombia. Hystrix—Ital J Mammal. 2016;27(2).

[90] Accordi I de A. Ecologia e conservação de aves em ambientes costeiros do Rio Grande do Sul. Universidade Federal do Rio Grande do Sul; 2008.

[91] Rosa A. Área de proteção ambiental do banhado grande: APABG: escolas, educação e preservação ambiental. Pontifícia Universidade Católica do Rio Grande do Sul; 2015.

[92] Kasper CB, Schneider A, Oliveira TG. Home range and density of three sympatric felids in the Southern Atlantic Forest, Brazil. Brazilian J Biol. 2016;76(1):228–32. 10.1590/1519-6984.19414 26871745

[93] Kasper CB, Mazim FD, Soares JBG, de Oliveira TG. Density estimates and conservation of Leopardus pardalis southernmost population of the Atlantic Forest. Iheringia Série Zool. 2015;105(3):367–71.

[94] Bolze GJ. Ecologia e comportamento de jaguatirica, Leopardus pardalis, no limite sul da Mata Atlântica. Universidade Federal do Rio Grande do Sul; 2019.

[95] Marques RV, Fábian ME. Daily activity patterns of medium and large neotropical mammals during different seasons in an area of high altitude Atlantic rain forest in the South of Brazil. Rev Bras Zoociências. 2018;19(3):38–64.

[96] Barrios-Garcia MN, Ballari SA. Impact of wild boar (Sus scrofa) in its introduced and native range: A review. Biol Invasions. 2012;14(11):2283–300.

[97] Ferreira GA, Nakano-Oliveira E, Genaro G. Domestic cat predation on Neotropical species in an insular Atlantic Forest remnant in southeastern Brazil. Wildlife Biol. 2014;20(3):167–75.

[98] Rocha-Mendes F, Bianconi G V. Opportunistic predatory behavior of margay, Leopardus wiedii (Schinz, 1821), in Brazil. Mammalia. 2009;73(2):151–2.

[99] Seibert JB, de O Moreira D, Mendes SL, Gatti A. Diet of two sympatric felids (Leopardus guttulus and Leopardus wiedii) in a remnant of Atlantic forest, in the montane region of Espírito Santo, southeastern Brazil. Bol do Mus Biol Mello Leitão. 2015;37(2):193–200.

[100] Amo L, Caro SP, Visser ME. Sleeping birds do not respond to predator odour. PLoS One. 2011;6(11). 10.1371/journal.pone.0027576 PMC3217974 22110676

[101] Massara RL, Paschoal AMO, Bailey LL, Doherty PF, Chiarello AG. Ecological interactions between ocelots and sympatric mesocarnivores in protected areas of the Atlantic Forest, southeastern Brazil. J Mammal. 2016;97(6):1634–44.

[102] Kiltie RA. Size ratio sympatric Neotropical cats. Oecologia. 1984;61:411–6. 10.1007/BF00379644 28311072

[103] Silva KVK de A, Kenup CF, Kreischer C, Fernandez FAS, Pires AS. Who let the dogs out? Occurrence, population size and daily activity of domestic dogs in an urban Atlantic Forest reserve. Perspect Ecol Conserv. 2018;16(4):228–33.

[104] Srbek-Araujo AC, Chiarello AG. Domestic dogs in Atlantic forest preserves of south-eastern Brazil: a camera-trapping study on patterns of entrance and site occupancy rates. Braz J Biol. 2008;68(4):771–9. 10.1590/s1519-69842008000400011 19197494

[105] Barnett B, Rudd R. Feral Dogs of the Galapágos Islands: Impact and Control. Int J Stud Anim Prob. 1983;4(1):44–58.

[106] de Almeida Curi NH, Araújo AS, Campos FS, Lobato ZIP, Gennari SM, Marvulo MFV, et al Wild canids, domestic dogs and their pathogens in Southeast Brazil: Disease threats for canid conservation. Biodivers Conserv. 2010;19(12):3513–24. 10.1007/s10531-010-9911-0 32214695 PMC7088301

[107] Krauze-Gryz D, Gryz JB, Goszczyński J, Chylarecki P, Zmihorski M. The good, the bad, and the ugly: space use and intraguild interactions among three opportunistic predators—cat (Felis catus), dog (Canis lupus familiaris), and red fox (Vulpes vulpes)- under human pressure. Can J Zool. 2012;90(12):1402–13.

[108] May SA, Norton TW. Influence of fragmentation and disturbance on the potential impact of feral predators on native fauna in Australian forest ecosystems. Wildl Res. 1996;23(4):387–400.

[109] Lacerda ACR, Tomas WM, Marinho-Filho J. Domestic dogs as an edge effect in the Brasília national park, Brazil: Interactions with native mammals. Anim Conserv. 2009;12(5):477–87.

[110] Horn JA, Mateus-Pinilla N, Warner RE, Heske EJ. Home range, habitat use, and activity patterns of free-roaming domestic cats. J Wildl Manage. 2011;75(5):1177–85.

[111] Campos CB, Esteves CF, Ferraz KMPMB, Crawshaw PG, Verdade LM. Diet of free-ranging cats and dogs in a suburban and rural environment, south-eastern Brazil. J Zool. 2007;273(1):14–20.

[112] Correa CMA, Braga RF, Louzada J, Menéndez R. Dung beetle diversity and functions suggest no major impacts of cattle grazing in the Brazilian Pantanal wetlands. Ecol Entomol. 2019;

[113] Schmutzer AC, Gray MJ, Burton EC, Miller DL. Impacts of cattle on amphibian larvae and the aquatic environment. Freshw Biol. 2008;53(12):2613–25.

[114] Marty JT. Effects of cattle grazing on diversity in ephemeral wetlands. Conserv Biol. 2005;19(5):1626–32.

[115] Chaikina NA, Ruckstuhl KE. The effect of cattle grazing on native ungulates: The good, the bad, and the ugly. Rangelands. 2006;28(3):8–14.

[116] Strassmann BI. Effects of cattle grazing and haying on wildlife conservation at National Wildlife Refuges in the United States. Environ Manage. 1987;11(1):35–44.

[117] Cruz F, Josh Donlan C, Campbell K, Carrion V. Conservation action in the Galàpagos: Feral pig (Sus scrofa) eradication from Santiago Island. Biol Conserv. 2005;121(3):473–8.

[118] Massei G, Genov P. The environmental impact of wild boar. Galemys Boletín Inf la Soc Española para la Conserv y Estud los mamíferos. 2004;16(1):135–45.

[119] Caruso N, Valenzuela AEJ, Burdett CL, Vidal EML, Birochio D, Casanave EB. Summer habitat use and activity patterns of wild boar Sus scrofa in rangelands of central Argentina. PLoS One. 2018;13(10):e0206513 10.1371/journal.pone.0206513 30356269 PMC6200264

