# Supplementary material for: Margay (Leopardus wiedii) in the southernmost Atlantic Forest: Density and activity patterns under different levels of anthropogenic disturbance
Source: PLoS One. 2020 May 6;15(5):e0232013. doi: 10.1371/journal.pone.0232013 (PMC7202647; doi:10.1371/journal.pone.0232013)
Supplement: S1 Table — (PDF) [file pone.0232013.s001.pdf]

S1 Table. Variance inflation factor test results of the density covariates models.

Variables	VIF score
Small mammals (smam)	2.08827
Small birds (sbirds)	1.550713
Distance water (diswater)	1.226475
Ocelot (ocelot)	1.645949
Vegetation cover (ndvi)	1.296269
Population density (popdens)	1.267173
Distance of roads (disroads)	1.802655
