# Supplementary material for: Margay (Leopardus wiedii) in the southernmost Atlantic Forest: Density and activity patterns under different levels of anthropogenic disturbance
Source: PLoS One. 2020 May 6;15(5):e0232013. doi: 10.1371/journal.pone.0232013 (PMC7202647; doi:10.1371/journal.pone.0232013)
Supplement: S2 Table — (PDF) [file pone.0232013.s002.pdf]

S2 Table. Total and independent records for small mammals, small birds, ocelot, domestic cat, domestic dog, wildboar, cattle, and humans.

Species	BPWR	PRO-MATA	TEUT	FNPF	Total
**Small mammals** (Small rodents and small marsupials)	10	16	120	143	289
**Small birds** (Columbiformes and Passeriformes)	1375	425	732	557	3089
**Ocelot** *(Leopardus pardalis)*	0	7	0	0	7
**Domestic cat** *(Felis catus)*	4	0	47	0	51
**Domestic dog** *(Canis lupus familiaris)*	20	10	39	6	75
**Wild boar** *(Sus scrofa)*	0	29	0	0	29
**Cattle (Cows and Horses** *(Bos taurus, Equus caballus)*	27	5	19	0	51
**Humans** *(Homo sapiens sapiens)*	13	50	8	1	72
