# Supplementary material for: Margay (Leopardus wiedii) in the southernmost Atlantic Forest: Density and activity patterns under different levels of anthropogenic disturbance
Source: PLoS One. 2020 May 6;15(5):e0232013. doi: 10.1371/journal.pone.0232013 (PMC7202647; doi:10.1371/journal.pone.0232013)
Supplement: S3 Table — (PDF) [file pone.0232013.s003.pdf]

S3 Table. Mean and standard deviation for time of sunrise and time ofsunset of the study period in each area; data obtained for the Viamão, São Franscisco de Paula, Teutônia and Passo Fundo municipalities (which include our study areas), RS, Brazil.

	Sunrise		Sunset	
	**Mean**	**SD**	**Mean**	**SD**
BPWR	06:20	0.01	19:35	0.024
PROMATA	06:36	0.009	20:20	0.002
TEUT	06:06	0.014	19:26	0.002
PFNF	06:43	0.014	19:45	0.029

*Data base collected from © 2011-2019 sunrise-and-sunset.com
